# Plasmid mediated penicillin and tetracycline resistance among *Neisseria gonorrhoeae* isolates from Kenya

**DOI:** 10.1186/s12879-020-05398-5

**Published:** 2020-09-25

**Authors:** Mary Wandia Kivata, Margaret Mbuchi, Fredrick Eyase, Wallace Dimbuson Bulimo, Cecilia Katunge Kyanya, Valerie Oundo, Wilton Mwema Mbinda, Willy Sang, Ben Andagalu, Olusegun O. Soge, Raymond Scott McClelland, John Distelhorst

**Affiliations:** 1 Institute for Biotechnology Research, Jomo Kenyatta University of Agriculture and Technology (JKUAT), P. O Box 62,000-00200, Thika, Kenya; 2 Department of Biological and Physical Science, Karatina University (KarU), P. O Box 1957-10101, Karatina, Kenya; 3 U.S. Army Medical Research Directorate-Africa, P. O Box 606, Village Market, Nairobi, 00621 Kenya; 4 Kenya Medical Research Institute (KEMRI), P. O Box 54840-00200, Nairobi, Kenya; 5 School of Medicine, Department of Biochemistry, University of Nairobi, P. O Box 30197, GPO, Nairobi, 00100 Kenya; 6 Department of Chemistry and Biochemistry, Pwani University, P. O Box 195-80108, Mombasa, Kenya; 7 Departments of Global Health and Medicine, University of Washington, 325 9th Avenue, Box 359931, Seattle, WA 98104 USA; 8 Departments of Medicine, Epidemiology, and Global Health, University of Washington, 325 9th Avenue, Box 359931, Seattle, WA 98104 USA

**Keywords:** *Neisseria gonorrhoeae*, Plasmid, *blaTEM*, *tetM*, Tetracycline, Penicillin

## Abstract

**Background:**

Treatment of gonorrhea is complicated by the development of antimicrobial resistance in *Neisseria gonorrhoeae* (GC) to the antibiotics recommended for treatment*.* Knowledge on types of plasmids and the antibiotic resistance genes they harbor is useful in monitoring the emergence and spread of bacterial antibiotic resistance. In Kenya, studies on gonococcal antimicrobial resistance are few and data on plasmid mediated drug resistance is limited. The present study characterizes plasmid mediated resistance in *N. gonorrhoeae* isolates recovered from Kenya between 2013 and 2018.

**Methods:**

DNA was extracted from 36 sub-cultured GC isolates exhibiting varying drug resistance profiles. Whole genome sequencing was done on Illumina MiSeq platform and reads assembled *de-novo* using CLC Genomics Workbench. Genome annotation was performed using Rapid Annotation Subsystem Technology. Comparisons in identified antimicrobial resistance determinants were done using Bioedit sequence alignment editor.

**Results:**

Twenty-four (66.7%) isolates had both β-lactamase (TEM) and TetM encoding plasmids. 8.3% of the isolates lacked both TEM and TetM plasmids and had intermediate to susceptible penicillin and tetracycline MICs. Twenty-six (72%) isolates harbored TEM encoding plasmids. 25 of the TEM plasmids were of African type while one was an Asian type. Of the 36 isolates, 31 (86.1%) had TetM encoding plasmids, 30 of which harbored American TetM, whereas 1 carried a Dutch TetM. All analyzed isolates had non-mosaic *penA* alleles. All the isolates expressing TetM were tetracycline resistant (MIC> 1 mg/L) and had increased doxycycline MICs (up to 96 mg/L). All the isolates had S10 ribosomal protein V57M amino acid substitution associated with tetracycline resistance. No relation was observed between PenB and MtrR alterations and penicillin and tetracycline MICs.

**Conclusion:**

High-level gonococcal penicillin and tetracycline resistance in the sampled Kenyan regions was found to be mediated by plasmid borne *blaTEM* and *tetM* genes. While the African TEM plasmid, TEM1 and American TetM are the dominant genotypes, Asian TEM plasmid, a new TEM239 and Dutch TetM have emerged in the regions.

## Background

Gonococcal infections are among the most predominant bacterial sexually transmitted infections (STI) worldwide. Accordingly, gonorrhea remains a major global health concern [1]. *N. gonorrhoeae* has over the years evolved and developed resistance to many of the antibiotics used to treat its infections including the penicillins and tetracyclines [1, 2]. The spread of these antibiotic resistance genes poses a challenge in treatment of gonococcal infections. Penicillins are β-lactam antibiotics that disrupt cell wall formation and integrity by targeting the major penicillin binding proteins (PBPs), mainly PBP1 and PBP2 (encoded by *ponA* and *penA* genes respectively) in gonococci [3, 4]. Tetracyclines inhibit the attachment of aminoacyl tRNA to the acceptor A site in the mRNA-ribosome complex by mainly binding to the 30S ribosomal subunit, and accordingly inhibiting protein synthesis [5]. In *N. gonorrhoeae*, resistance to both penicillin and tetracycline is mediated through two mechanisms: chromosomal mutations and acquisition of plasmid borne genes, mainly *blaTEM-1* for penicillin and *tetM* for tetracycline [6–9].

Seven types of plasmids harboring β-lactamase have been described in penicillinase-producing *Neisseria gonorrhoeae* (PPNG) and named based on geographical areas where they were first described as: Asian; African; Rio/Toronto; Nimes; Johannesburg; New Zealand and Australian [10–15]. The Asian type is the ancestral plasmid from which either deletions or insertions gave rise to the other six plasmid types [12, 16]. These plasmids have been shown to carry *blaTEM-1* encoding TEM1 β-lactamase or its derivatives [17]. TEM1 β-lactamase destroys the activity of β-lactam drugs by hydrolyzing the amide bond in the β-lactam ring but it is not active against extended-spectrum cephalosporins [18]. Single Nucleotide Polymorphisms (SNPs) in *blaTEM-1* resulting in alteration of amino acid configuration around TEM1 β-lactamase active site can convert it to an extended spectrum β-lactamase (ESBL) [19]. The ESBL are more stable and potent and can breakdown cephalosporins including ceftriaxone, the last first line monotherapy for treatment of gonorrhea. *blaTEM-135* encoding a more stable TEM-135 β-lactamase which differs from TEM1 β-lactamase by one amino acid substitution (M182T) has been described in gonococci from several countries [17, 20–22]. It has been described as an intermediate between TEM1 β-lactamase and extended broad spectrum β-lactamase [23]. Both *blaTEM-1* and *blaTEM-135* have mainly been described in Asian, African and Toronto plasmid types and associated with epidemic outbreaks [16, 24, 25].

Chromosomal modifications in at least five different genes including *penA*, *ponA*, *mtrR*, *porB*, and *pilQ* have been implicated in chromosomally mediated gonococcal penicillin resistance [26]. Modifications in *penA,* and *ponA* alter the three dimensional structures of PBP2 and PBP1. This reduces the affinity of PBPs for penicillin and consequently reduces susceptibility to β-lactams [27]. Recombination of gonococcal *penA* with *penA* genes of commensal N*eisseria* species has led to development of a mosaic-like *penA* structure, which has been associated with resistance to cefixime and ceftriaxone in gonococci from different regions [28–31].

The *mtrR* gene encodes MtrR which represses the expression of the multiple transferrable resistance CDE (MtrCDE) efflux pump [26]. In *N. gonorrhoeae*, mutations in the *mtrR* promoter or the MtrR encoding region lead to over expression of the MtrCDE efflux pump and has been associated with resistance to antibacterial agents [32]. Reduced drug permeation resulting from modifications of porinB (PorB also referred to as PenB alterations) encoded by *porB* has also been associated with an intermediate-level resistance to both penicillin and tetracycline in *N. gonorrhoeae* [33].

High tetracycline resistance in gonoccoci is mediated by a transposon-borne (Tn916) class M tetracycline (TetM) resistance determinant. TetM binds to 30S ribosomal subunit thereby blocking tetracycline from binding to its target [5, 34]. There are two different TetM determinants; American and Dutch which are carried by either of two 25.2 MDa conjugative plasmids named “American” and “Dutch” type plasmids found in gonococci [35, 36]. Chromosomal modifications which mediate tetracycline resistance in gonococci include: a) V57M amino acid substitution in S10, a 30S ribosomal protein encoded by *rpsJ* gene. This modification results in an altered tetracycline binding site and consequently reduced binding affinity, and b) modifications in *mtrR* and *porB* which result in reduced drug accumulation [37].

In Kenya a few studies have reported penicillin and tetracycline resistance in *N. gonorrhoeae* since the 1970s [38–41]. Following these reports the use of both penicillin and tetracycline for treatment of gonococcal infections was stopped [42]. Nevertheless, the two drugs are widely available to the public and are inappropriately used through self prescription in many parts of Africa including Kenya [43–45].

Determining the plasmid types and characterizing the antibiotic resistance genes they harbor is significantly important. It helps in monitoring the emergence and spread of antibiotic resistant *N. gonorrhoeae* isolates as well as the spread of plasmid borne genes between different bacteria. Poor surveillance and the fact that both penicillin and tetracycline are neither the first nor the second line drug of choice for treatment of gonorrhea, has limited data on plasmid types and plasmid borne resistance genes in Kenyan gonococci. This study therefore sought to determine the prevalence and identity of TEM plasmids types. We also, characterized both TEM and TetM encoding genes in Kenyan *N. gonorrhoeae* isolates recovered from heterosexual population between 2013 and 2018.

## Methods

### Bacterial isolates and antimicrobial susceptibility testing

Study isolates were obtained as part of an ongoing STI surveillance study (WRAIR#1743, KEMRI#1908) under Armed Forces Health Surveillance at the US Army Medical Research Directorate-Africa (USAMRD-A). The isolates were recovered from both urethral and endocervical samples obtained from male and female patients seeking treatment in selected clinics from four geographic locations in Kenya (Nairobi, Coastal Kenya, Nyanza, and Rift Valley) between 2013 and 2018. Frozen isolates were thawed and inoculated on GC agar base supplemented with vancomycin, nystatin, colistin and trimethoprim lactate, 1% IsoVitaleX (Becton Dickinson, US) and 10% Hemoglobin solution (Becton Dickinson, US) and incubated at 37 °C in 3–5% CO_2_ for 18–24 h. *N. gonorrhoeae* was confirmed through colony morphology, Gram stain, oxidase, catalase, and APiNH® (Biomerieux) biochemical tests prior to antimicrobial susceptibility testing and DNA extraction. 0.5 MacFarland standard GC inoculums were inoculated on GC agar base medium (Becton Dickinson, US) supplemented with 1% IsoVitaleX (Becton Dickinson, US) and 10% Hemoglobin solution (Becton Dickinson, US). Minimum inhibitory concentrations (MICs) of ceftriaxone; cefixime; azithromycin; ciprofloxacin; norfloxacin; spectinomycin; tetracycline; doxycycline; penicillin and gentamicin were determined using E-test® (Biomerieux) method according to manufacturer’s instructions [46, 47]. WHO K and WHO O reference gonococcal strains [48] (antimicrobial susceptibility patterns described in Table 1 below) were used to ensure accuracy of AST data.

**Table 1:** Antimicrobial susceptibility patterns of the WHO reference strains (mg/L) [48]

Antimicrobial	WHO K	WHO O
Ceftriaxone	0.064	0.032
Cefixime	0.25	0.016
Azithromycin	0.25	0.25
Ciprofloxacin	> 32	0.008
Spectinomycin	16	> 1024
Tetracycline	2	2
Penicillin	2	> 32
β-lactamase (PPNG)	Negative	Positive

***PPNG*** **Penicillinase producing** ***N. gonorrhoeae*. Currently there are no EUCAST MIC breakpoints for norfloxacin, gentamycin and doxycycline for** ***N. gonorrhoeae***

MICs breakpoints were interpreted with reference to European Committee on Antimicrobial Susceptibility Testing (EUCAST) version 8.0, 2018 standards (http://www.eucast.org/fileadmin/src/media/PDFs/EUCAST_files/Breakpoint_tables/v_8.0_Breakpoint_Tables.pdf) as shown in Table 2. Thirty six viable *N. gonorrhoeae* isolates exhibiting varying antibiotic resistance profiles were chosen for analysis (Table 3).

**Table 2:** EUCAST breakpoints used for MIC interpretation for the tested antibiotics

Antibiotic	MIC breakpoint (mg/L)	
	Susceptible ≤	Resistant >
Ceftriaxone	0.125	0.125
Cefixime	0.125	0.125
Azithromycin	0.25	0.5
Ciprofloxacin	0.03	0.06
Spectinomycin	64	64
Tetracycline	0.5	1
Penicillin	0.06	1

### DNA extraction

Both Genomic and plasmid DNA were extracted using QIAamp DNA Mini Kit and QIAprep Spin Miniprep Kit (QIAGEN, Hilden, Germany) “respectively” according to the manufacturer’s instructions. Qubit dsDNA HS Assay was used to quantitate DNA using Qubit 3.0 fluorometer, (Thermo Fisher Scientific Inc. Wilmington, Delaware USA) according to the manufacturer’s instructions, and DNA stored at − 20 °C prior to sequencing.

### Whole-genome sequencing and sequence analysis

Illumina Nextera XT kit (Illumina Inc. San Diego, CA, USA) was used to prepare libraries from 1 ng of genomic DNA of each sample as per manufacturer’s instructions. Sequence reads were generated on Illumina MiSeq platform (Illumina, San Diego, CA, USA) using a paired-end 2 × 300 bp protocol [49]. The generated reads are linked to NCBI BioProjects: PRJNA481622 and PRJNA590515. Raw reads were trimmed for quality and assembled de novo using CLC Genomics Workbench version 12.0. Blast searches were performed using BLASTN suite in National Center for Biotechnology Information (NCBI) (https://www.ncbi.nlm.nih.gov). Assembled genomes were annotated in Pathosystems Resource Integration Center version 3.5.31 (PATRIC) (https://www.patricbrc.org) using Rapid Annotation Subsystem Technology (RAST) [26, 50]. Identified TEM and TetM determinants were downloaded and compared with reference TEM1 (GenBank Accession number WP_000027057.1) and TetM (GenBank Accession number WP_047922456.1) downloaded from the NCBI website (https://www.ncbi.nlm.nih.gov). Identification and comparison of amino acid alterations in antimicrobial resistance determinants known to confer drug resistance in *N gonorrhoeae* were done using Bioedit sequence alignment editor version 7.0.5 [51].

### Statistical analysis

Wilcoxon Mann-Whitney statistical tests were conducted in GraphPad Prism version 7.0.4 (www.graphpad.com). Two tailed statistical comparisons were performed with significance level set at *P* < 0.05.

## Results

### Antibiotic susceptibility patterns of the study isolates

Of the 36 analyzed isolates 26 (72.2%) were penicillin resistant (MICs > 1 mg/L), whereas 31 (86.1%) were tetracycline resistant (MICs> 1 mg/L). Thirty-four (94.4%) isolates were ciprofloxacin resistant (MICs > 0.06). Low level of azithromycin resistance (MICs 1-2 mg/L) was observed in 3 (8.3%) isolates. None of the isolates was resistant to cefixime, ceftriaxone, or spectinomycin (Table 3). Twenty-four (66.7%) isolates were resistant to both penicillin and tetracycline, while 23 (63.9%) isolates were resistant to penicillin, tetracycline and ciprofloxacin. Two of the three isolates expressing low level azithromycin resistance were also resistant to penicillin, tetracycline and ciprofloxacin.

**Table 3:** Antimicrobial susceptibility data of the study isolates

Isolate details		MICs (mg/L)						
Sample ID	Year of isolation	CFX	CRO	PEN	TET	CIP	AZM	SPT
KNY_NGAMR1	2015	< 0.016	< 0.002	0.064	0.064	0.38	0.25	8
KNY_NGAMR2	2015	< 0.016	< 0.002	64	0.5	0.016	0.5	8
KNY_NGAMR3	2015	< 0.016	NT	48	16	3	0.125	6
KNY_NGAMR4	2016	< 0.016	< 0.002	8	12	0.006	0.125	2
KNY_NGAMR5	2016	< 0.016	0.006	3	16	4	0.125	2
KNY_NGAMR6	2017	< 0.016	< 0.016	0.094	0.75	12	0.25	3
KNY_NGAMR7	2014	< 0.016	0.008	> 256	16	8	2	16
KNY_NGAMR8	2013	< 0.016	0.002	0.064	0.5	3	0.25	4
KNY_NGAMR10	2016	< 0.016	< 0.016	> 256	32	8	0.38	16
KNY_NGAMR11	2016	< 0.016	< 0.016	12	12	3	0.25	8
KNY_NGAMR13	2014	< 0.016	< 0.002	8	12	3	0.125	24
KNY_NGAMR14	2015	< 0.016	< 0.002	64	24	16	0.125	8
KNY_NGAMR15	2014	< 0.016	< 0.016	48	32	4	0.5	12
KNY_NGAMR16	2015	< 0.016	< 0.016	0.19	4	8	0.38	4
KNY_NGAMR17	2015	< 0.016	< 0.016	0.38	16	6	0.5	6
KNY_NGAMR18	2015	< 0.016	< 0.016	0.5	24	0.38	0.5	6
KNY_NGAMR19	2015	< 0.016	0.094	0.19	64	4	1.5	1
KNY_NGAMR20	2015	< 0.016	0.004	12	24	8	0.125	12
KNY_NGAMR21	2016	< 0.016	< 0.016	32	24	3	0.125	12
KNY_NGAMR22	2016	< 0.016	0.004	2	32	4	1	12
KNY_NGAMR23	2017	< 0.016	< 0.002	12	8	12	0.125	4
KNY_NGAMR24	2014	< 0.016	< 0.016	2	16	4	0.38	8
KNY_NGAMR26	2016	< 0.016	< 0.016	8	32	1.5	0.25	8
KNY_NGAMR27	2016	< 0.016	< 0.016	1	32	24	0.25	8
KNY_NGAMR28	2017	< 0.016	< 0.002	96	32	24	0.5	8
KNY_NGAMR29	2017	< 0.016	< 0.002	0.094	12	12	0.25	2
KNY_NGAMR30	2017	< 0.016	< 0.002	12	12	4	0.125	8
KNY_NGAMR31	2017	< 0.016	< 0.002	0.38	48	> 32	0.25	3
KNY_NGAMR32	2017	< 0.016	< 0.002	8	0.5	16	0.047	1.5
KNY_NGAMR33	2016	< 0.016	< 0.016	3	32	2	0.25	4
KNY_NGAMR35	2013	NT	NT	> 256	48	32	0.125	NT
KNY_NGAMR41	2018	< 0.016	< 0.016	32	12	16	NT	4
KNY_NGAMR42	2018	< 0.016	< 0.016	32	24	6	0.125	8
KNY_NGAMR50	2018	< 0.016	< 0.016	16	16	1.5	0.064	4
KNY_NGAMR53	2018	< 0.016	< 0.016	6	6	3	0.19	4
KNY_NGAMR54	2018	< 0.016	< 0.016	6	2	2	0.125	6

*CRO* Ceftriaxone, *CFX* Cefixime, *PEN* Penicillin, *SPT* Spectinomycin, *AZM* Azithromycin, *CIP* Ciprofloxacin, *TET* Tetracycline, *NT* Not tested

### Plasmid types and prevalence

Twenty-four of the 36 (66.7%) isolates had both TEM and TetM encoding plasmids whereas 3 (8.3%) isolates lacked plasmids. Two of these three isolates were penicillin susceptible (0.064 mg/L) while one had intermediate penicillin resistance (0.094 mg/L). The isolates had intermediate to susceptible tetracycline MICs (0.5–0.75 mg/L) (Table 6). Of the 36 isolates, 26 (72.2%) harbored TEM encoding plasmids and were therefore PPNG (Table 4). Twenty-five (96.2%) of the PPNG had the African type plasmid (pDJ5) while 1 (3.8%), had an Asian type plasmid (pDJ4) (Table 5). Thirty one (86.1%) of the 36 isolates harbored TetM encoding plasmids (Table 4). Two (5.5%) PPNG lacked TetM encoding plasmid while seven (19.4%) GC harboring TetM encoding plasmids lacked TEM encoding plasmid (Table 4).

**Table 4:** β-lactamase encoding and TetM encoding plasmids

	β-lactamase encoding	Non-β-lactamase encoding	Totals
TetM encoding	24 (66.7%)	7 (19.4%)	**31** (86.1%)
Non-TetM encoding	2 (5.5%)	3 (8.3%)	**5** (13.9%)
**Total**	**26** (72.2%)	**10** (27.8%)	**36** (100%)

**Table 5:** TEM and TetM genotypes and Plasmid prevalence

PPNG plasmid type	African, 25 (96.2%)	Asian, 1 (3.8%)	26 (100%)
TetM	American, 30 (96.8%)	Dutch, 1 (3.2%)	**31** (100%)
TEM genotype	*blaTEM-1*, 21 (81.5%)	*blaTEM-239*, 5 (18.5%)	**27 (100%)**

### TEM and TetM genotypes

Of the 26 PPNG, 21 (80.8%) expressed TEM1 β-lactamase encoded by *blaTEM-1* gene, while 5 (19.2%) isolates expressed a β-lactamase encoded by a recently described *blaTEM* allele (NEIS2357 allele 10) (Table 5) [52]. All these new TEM1 alleles were carried by African type TEM plasmids. The sequence of the allele was deposited under GenBank accession number MK497256l and assigned as class A β-lactamase TEM239 (*blaTEM*) gene, *blaTEM-239* allele with a protein accession number QBC36181. American TetM determinant was identified in 30 (96.8%) of the 31 isolates harboring a TetM plasmid, whereas Dutch TetM determinant was identified in only one (3.2%) of those isolates (Table 5).

### Correlation between TEM and TetM presence and penicillin and tetracycline susceptibility

**Fig. 1:**
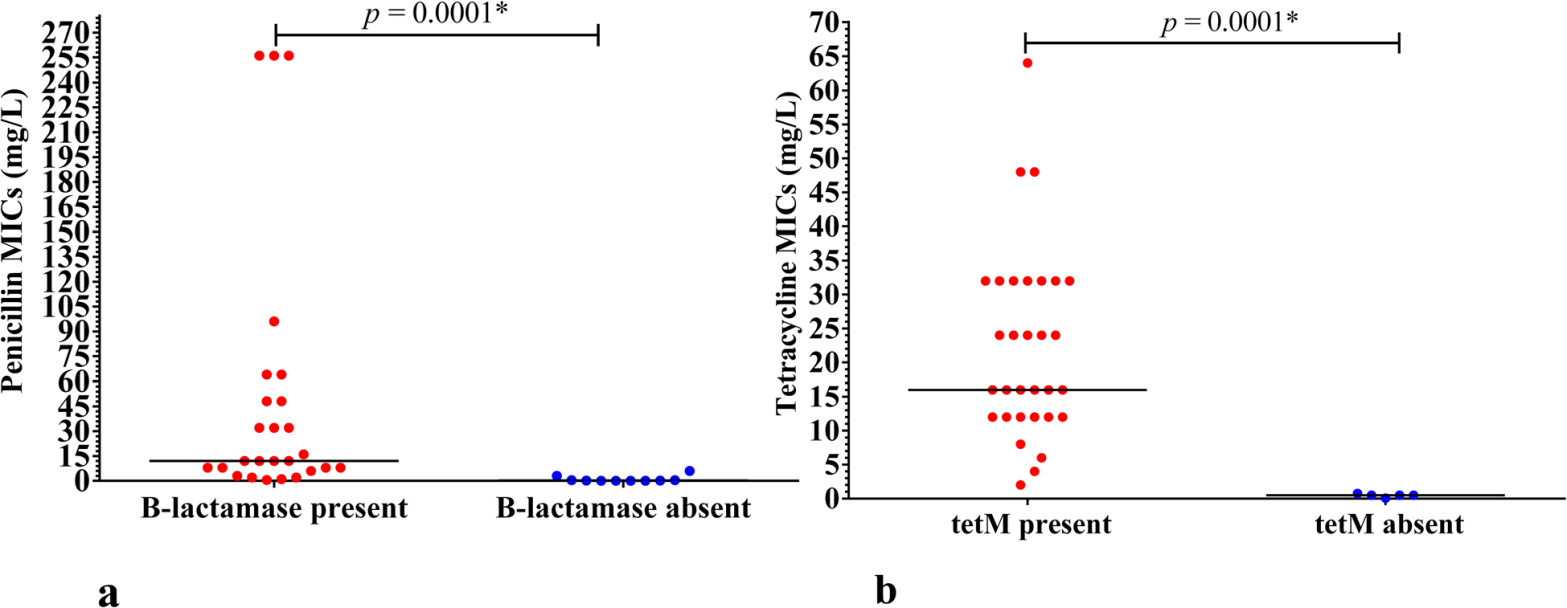
This figure shows the relationship between a) penicillin susceptibilities and the presence of β-lactamase, and b) tetracycline susceptibilities and the presence and tetM. The central bar across each group of points locates the median for that group.

The PPNG had significantly high penicillin MICs (Median 12.00, inter-quartile range (IQR) 44.5), compared to the non-PPNG strains (Median 0.1900, IQR 0.9485, *p* = 0.0001*) (Fig. 1a). Twenty-four (92.3%) of the 26 PPNG were penicillin resistant with MICs > 1 mg/L, while the remaining two PPNG (KNY_NGAMR18 and KNY_NGAMR27) had intermediary penicillin susceptibility (MICs> 0.06–1 mg/L) (Table 6). Two isolates, KNY_NGAMR33 and KNY_NGAMR54 which were non-PPNG, had penicillin resistant MIC values of 3 and 6 mg/L, respectively (Table 6). All isolates expressing TetM had significantly high tetracycline MICs (Median 16.00, IQR 20) compared to the non-TetM expressing isolates (Median 0.5000, IQR 0.343, *p* = 0.0001*) and were all tetracycline resistant (MICs> 1 mg/L) (Fig. 1b). Furthermore, all the isolates harboring TetM had higher doxycycline MICs (up to 96 mg/L). The isolate expressing a Dutch TetM had the highest doxycycline and tetracycline MICs (96 mg/L and 64 mg/L respectively) (Table 6).

**Table 6:** Penicillin and tetracycline antimicrobial susceptibility data and identified AMR determinants

		Tetracyclines				Penicillin G					
		MIC (mg/L)		tetM Plasmid	S10	MIC (mg/L)	β-lactamase	***penA*** allele	PonA	MtrR	PenB
	Sample ID	DOX	TET			PEN					
1	KNY_NGAMR1	0.75	0.064	*Absent*	V57M	0.064	NP	Pattern XIV	–	–	A121G, G120D, −N122
2	KNY_NGAMR2	1.5	0.5	*Absent*	V57M	64	TEM1^a^	Pattern II	L421P	G45D, −A13	G120D
3	KNY_NGAMR3	6	16	Present	V57M	48	TEM239	Pattern IX	L421P	A39T	–
4	KNY_NGAMR4	16	12	Present	V57M	8	TEM1	Pattern XIX	–	A39T	A121S, N122K
5	KNY_NGAMR5	12	16	Present	V57M	3	TEM1	Pattern IX	L421P	H105Y	A121G, G120N, −N122
6	KNY_NGAMR6	0.75	0.75	*Absent*	V57M	0.094	NP	Pattern II	–	A39T	–
7	KNY_NGAMR7	12	16	Present	V57M	> 256	TEM239	Pattern XXII	L421P	–	A121S, N122K
8	KNY_NGAMR8	0.5	0.5	*Absent*	V57M	0.064	NP	Pattern XIV	L421P	A39T	A121G, G120D, −N122
9	KNY_NGAMR10	24	32	Present	V57M	> 256	TEM1	Pattern II	–	A39T	–
10	KNY_NGAMR11	12	12	Present	V57M	12	TEM1	Pattern XIV	L421P	A39T	A121G, G120D, −N122
11	KNY_NGAMR13	12	12	Present	V57M	8	TEM1	Pattern XIV	L421P	A39T	A121G, G120D, −N122
12	KNY_NGAMR14	8	24	Present	V57M	64	TEM1	Pattern II	–	A39T	A121S, N122K
13	KNY_NGAMR15	16	32	Present	V57M	48	TEM1	Pattern XIV	L421P	A39T	A121G, −N122
14	KNY_NGAMR16	8	4	Present	V57M	0.19	NP	Pattern XIV	–	A39T	A121G, −N122
15	KNY_NGAMR17	12	16	Present	V57M	0.38	NP	Pattern XXII	L421P	T86A, D79N, H105Y	–
16	KNY_NGAMR18	32	24	Present	V57M	0.5	TEM1	Pattern XIX	–	A39T	–
17	KNY_NGAMR19	96	64	Present^b^	V57M	0.19	NP	Pattern XIV	L421P	A39T	–
18	KNY_NGAMR20	16	24	Present	V57M	12	TEM1	Pattern IX	L421P	A39T	A121G, G120D, −N122
19	KNY_NGAMR21	16	24	Present	V57M	32	TEM1	Pattern XIV	L421P	A39T	A121G, G120D, −N122
20	KNY_NGAMR22	12	32	Present	V57M	2	TEM1	Pattern XIX	–	A39T	–
21	KNY_NGAMR23	16	8	Present	V57M	12	TEM1	Pattern IX	L421P	A39T	A121G, G120D, −N122
22	KNY_NGAMR24	16	16	Present	V57M	2	TEM1	Pattern XIX	–	A39T	–
23	KNY_NGAMR26	24	32	Present	V57M	8	TEM239	Pattern XIV	–	A39T	A121G, −N122
24	KNY_NGAMR27	16	32	Present	V57M	1	TEM239	Pattern XIV	–	A39T	–
25	KNY_NGAMR28	24	32	Present	V57M	96	TEM1	Pattern II	–	A39T	A121G, −N122
26	KNY_NGAMR29	4	12	Present	V57M	0.094	NP	Pattern XIV	L421P	A39T	A121G, −N122
27	KNY_NGAMR30	12	12	Present	V57M	12	TEM1	pattern XIV	L421P	A39T	A121G, −N122
28	KNY_NGAMR31	24	48	Present	V57M	0.38	NP	Pattern II	–	A39T	A121G, G120D, −N122
29	KNY_NGAMR32	1	0.5	*Absent*	V57M	8	TEM1	Pattern XIV	L421P	A39T	A121G, G120D, −N122
30	KNY_NGAMR33	24	32	Present	V57M	3	NP	Pattern XIV	–	A39T	A121G, −N122
31	KNY_NGAMR35	24	48	Present	V57M	> 256	TEM1	Pattern II	–	A39T	–
32	KNY_NGAMR41	12	12	Present	V57M	32	TEM1	Pattern XIX	–	A39T	–
33	KNY_NGAMR42	12	24	Present	V57M	32	TEM1	Pattern XIV	–	T86A, D79N, H105Y	–
34	KNY_NGAMR50	32	16	Present	V57M	16	TEM1	Pattern II	–	A39T	A121G, G120D, −N122
35	KNY_NGAMR53	16	6	Present	V57M	6	TEM239	Pattern XIV	L421P	A39T	A121G, −N122
36	KNY_NGAMR54	8	2	Present	V57M	6	NP	Pattern XXII	–	A39T	–

***DOX*** **Doxycycline,** ***TET*** **Tetracycline,** ***PEN*** **Penicillin** ^**(**a**)**^**β-lactamase encoding plasmid with an Asian plasmid backbone,** ^**(**b**)**^**Dutch TetM determinant,** ***NP*** **Not present, “-” no mutation identified, (−N122)- deletion of N at position 122 of PenB, (−A13)- Adenine deletion in the 13 bp inverted repeat region between the − 10 and − 35 hexamers of the** ***mtrR*** **promoter**

**Pattern XXII;** D346, F505L, A511V, A517G H542N, P553V, K556Q, I557V, I567V, N575, A576V

**Pattern IX;** D346, F505L, A511V, A517G, P552L

**Pattern XIX;** D346, F505L, A511V, A517G, H542N, I567V, N575, A576V

**Pattern XIV;** D346, F505L, A511V, A517G, H542N

**Pattern II;** D346, F505L, A511V, A517G

### Chromosomally encoded antimicrobial resistance determinants in the analyzed isolates

All isolates had non-mosaic *penA* alleles which have been associated with penicillin resistance in gonococci [27]. Five different non-mosaic PenA patterns were identified; patterns XXII, IX, XIX, XIV, and II [53]. Pattern IX was only identified in penicillin resistant isolates. Pattern XIV which was identified in 16 PPNG was the most prevalent (61.5%) (Table 6).

Reduced drug accumulation resulting from reduced drug influx (due altered or lost porins) or active efflux pump has been shown to contribute/produce additive effects to drug resistance in *N. gonorrhoeae* [54, 55]. Five different patterns of PenB were identified in 23 (63.9%) study isolates: pattern I (G120D); pattern II (A121G, G120D, and -N122); pattern III (A121G, G120N, and -N122); pattern IV (A121S, and N122K) and pattern V (A121G, and -N122) (Table 6). These patterns were formed by alterations in PenB that have been associated with reduced drug accumulation and consequently drug resistance in gonococci [56]. There was no significant increase in both penicillin (Median 8.000, IQR 29, *p* = 0.6779) and tetracycline (Median 12.0000, IQR 18, *p* = 0.1203) MICs observed in isolates harboring the above described PenB amino acid changes when compared to the isolates without the PenB alterations (penicillin; Median 2.000, IQR 39.5600, tetracycline; Median 24.0000, IQR 18).

Modifications in the MtrR promoter and encoding gene which have previously been associated with antibiotic resistance in gonococci were identified in 34 (94.4%) of the 36 analyzed isolates [32]. The modifications included: Deletion of Adenine in the 13 bp (−A13) inverted repeat region between the − 10 and − 35 hexamers of the *mtrR* promoter (1 isolate); G45D (1 isolate); A39T (30 isolates); T86A (2 isolates); D79N (2 isolates) and H105Y (3 isolates) (Table 6). One of the two isolates lacking *mtrR* modifications (KNY_NGAMR7) expressed both TEM239 and TetM and was penicillin and tetracycline resistant. The remaining isolate (KNY_NGAMR1) that lacked MtrR modifications lacked both TEM and TetM. It was both penicillin and tetracycline susceptible. Only two isolates expressed both T86A and D79N substitutions in addition to H105Y. One of these two isolates was a penicillin resistant PPNG while the other was a non-PPNG and had intermediary penicillin susceptibility. They both expressed TetM and were tetracycline resistant. Both -A13 and G45D were expressed by only 1 isolate, KNY_NGAMR2 which was a penicillin resistant PPNG. This isolate lacked TetM and was tetracycline susceptible. A39T the most prevalent modification (83.3%), was not expressed concurrently with any other MtrR modification (Table 6). No significant increase in both penicillin (Median 8.000, IQR 31.1250, *p* = 0.8446 and tetracycline (Median 16.0000, IQR 21, *p* = 0.2542) MICs were observed in the isolates expressing MtrR A39T substitution when compared to the isolates without the MtrR A39T substitution (penicillin; Median 17.5000, IQR 111.6990, tetracycline; Median 16.0000, IQR 17.6090).

L421P amino acid substitution associated with decreased rate of penicillin acylation in gonococci was identified in 17 (47.2%) of the 36 analyzed isolates (Table 6). There was no significant increase in penicillin MICs observed in the isolates harboring PonA L421P amino acid changes (Median 12.00, IQR 38.31, *p* = 0.7124) when compared to isolates expressing wild type PonA (Median 6.000, IQR 31.5000). PilQ E666K substitution associated with penicillin resistance was not observed in the present study [27].

One of the two penicillin resistant non-PPNGs, KNY_NGAMR33 expressed altered PenA, MtrR and PenB while KNY_NGAMR54 expressed altered PenA, and MtrR (Table 6). The two PPNG isolates which had intermediary penicillin susceptibility both lacked PonA and PenB alterations but harbored A39T MtrR substitution.

V57M substitution in S10 ribosomal proten, together with *mtrR* and *penB* mutations have been shown to increase tetracycline resistance [37]. S10 V57M was identified in all isolates both tetracycline susceptible and resistant. Three of five isolates with susceptible to intermediary tetracycline susceptibility had both MtrR and PenB amino acid changes while two had either MtrR or PenB amino acid changes each (Table 6). Other chromosomally encoded antimicrobial determinants identified in the analyzed isolates included previously reported altered GyrA (S91F and D95G/A) and ParC (E91G and S87R) which confer resistance to fluoroquinolones [57]. m*efA/E* genes encoding a membrane bound efflux MefA protein, rRNA methylase encoding *erm (B/C/F)* genes, and mutations in 23S ribosomal RNA and large subunit ribosomal proteins L4 encoded by *rplD* and L22 encoded by *rplV* all known to confer resistance to macrolides were not identified in any isolate [58–61]. Two of the three isolates which had a low level azithromycin resistance, expressed A39T MtrR modification, while one expressed an altered PenB (Table 6). Mutations in 16S ribosomal RNA and small subunit ribosomal protein S5 encoded by *rpsE* which confer resistance to spectinomycin [62, 63] were also not found in any of the isolates were also not found in any of the isolates were also not found in any of the isolates. Mosaic *penA* alleles, associated with increased and resistant cefixime and ceftriaxone MICs in gonococci were not identified in the present study.

## Discussion

Two β-lactamase plasmid types of different origins (African and Asian) were identified in this study. African β-lactamase plasmid (pDJ5) which was first identified from Africa [16, 64] was predominant. These findings are similar to observations of a previous study from Coastal Kenya [52]. The Asian type β-lactamase plasmid (pDJ4) initially described in Asia has been associated with epidemic outbreaks in Asian countries [12]. Five of the PPNG harbored a unique *blaTEM-239* allele which has only been reported in Kenya by a previous study [52]. In the study that unraveled *blaTEM-239*, high level penicillin resistance was associated with the allele [52]. However, from our findings, one isolate expressing TEM239 had an MIC suggesting intermediate susceptibility to penicillin while four were resistant.

Although a significant association was observed between penicillin MICs and the presence of TEM, this study also found two non-PPNG isolates which were penicillin resistant. These two isolates expressed chromosomal modifications which have previously been associated with penicillin resistance. These findings indicate that resistance to penicillin in the analyzed Kenyan gonococci is also mediated by chromosomal modifications mechanisms in addition to the plasmid borne TEM. β-lactamase production in gonococci has been associated with resistant penicillin MICs [65] [66]. Contrary to these previous findings, this study identified two PPNG isolates which were not resistant to penicillin. They both lacked PonA and PenB alterations but expressed A39T MtrR substitution known to mediate penicillin resistance in gonococci. Continued surveillance and monitoring of β-lactamase production is required in order to understand the susceptibility pattern observed in these two non-penicillin resistant PPNGs.

The predominance of the American TetM in the present study confirms the expected epidemiology of this resistance marker. In a study by Turner et al., the American TetM was identified in 14 GC isolates with a Kenyan origin [67], suggesting that the American TetM originated from equatorial regions of Africa. The American type was also found to be predominant in GC isolates from the United Kingdom, and eastern and central Africa [68]. Thus, the findings of this study agree with these previous studies. Dutch type TetM is predominant in GC isolates from the Netherlands, Asia and South America [68]. In the present study, identification of a Dutch type TetM in one of the study isolate shows that there is an introduction of the GC expressing Dutch TetM determinant into the sampled Kenyan regions.

Chromosomal V57M substitution in S10 modulates the affinity of tetracycline for its 30S ribosomal target and together with *mtrR* and *penB* mutations, have been shown to cause chromosomally mediated tetracycline resistance in gonococci (MIC≥2 mg/L) [26, 37]. A study from Coastal Kenya observed S10 V57M substitution in both tetracycline resistant and susceptible gonococcal isolates and suggested that this substitution had no effect on the observed low or high level tetracycline resistance [52]. In the present study all the isolates both tetracycline resistant and susceptible had the S10 V57M substitution. Increased and tetracycline resistant MICs were observed only in isolates expressing TetM protein, indicating that resistance to tetracycline in the analyzed Kenyan gonococci is mainly plasmid mediated. Contrary to the association of TetM with high-level tetracycline resistance (MIC≥16 mg/L) in gonococci, we observed ten tetM expressing isolates which had a lower level of tetracycline resistance (MIC range of 2-12 mg/L) (Table 6) [69]. High doxycycline MICs were also observed in isolates which harbored TetM. This observation is similar to the findings of the former Coastal Kenya study and indicates that TetM is involved in mediating doxycycline resistance in the analyzed Kenyan gonococci [52].

A previous study by Sun et al., (2010) reported novel PorB deletions at both A121 and N122 positions, and associated these changes with high levels of both chromosomal penicillin (MIC of 4–8 mg/L) and tetracycline (MICs of 4–16 mg/L) resistance [56]. It is worth noting that the present study identified deletion at only position N122. Additionally, non-tetM and non-PPNG isolates harboring N122 PorB deletions did not have high penicillin (0.064-3 mg/L) and tetracycline (0.064–0.5 mg/L) MICs as reported in the previous study. Comparing our findings with those of the previous study suggests that for such high levels of both chromosomal penicillin and tetracycline resistance to occur the deletions at A121 and N122 have to occur concurrently.

The identification of an Asian type β-lactamase plasmid and Dutch TetM determinant in Kenyan *N*. *gonorrhoeae* isolates indicates that there is circulation of plasmid mediated antibiotic resistance between different *N*. *gonorrhoeae* isolates from different countries. Although the use of penicillin and tetracycline for gonorrhea treatment was stopped many years ago, they are widely available to the public and are inappropriately used through self prescription in many parts of Africa [43, 44]. The observed high prevalence of plasmid mediated penicillin and tetracycline resistance indicates that these drugs are not suitable for gonorrhea treatment.

Mosaic *penA* alleles shown to confer resistance to extended-spectrum cephalosporins in gonococci were not observed in the present study [29–31]. These findings correlate with the observed phenotypic patterns as all the analyzed isolates were susceptible to both ceftriaxone and cefixime. This study did not identify any antimicrobial resistance determinants specifically associated with macrolide resistance. These finding explains the azithromycin phenotypes observed in the study isolates where a larger proportion of the study isolates were azithromycin susceptible with only 8.3% of the isolates having a low level azithromycin resistance. The observed low level azithromycin resistance could be mediated by reduced drug accumulation resulting from modified PenB and MtrR which were identified in these isolates. Absence of molecular markers specifically associated with high levels of azithromycin, cefixime and ceftriaxone in the present study, shows that these antibiotics are still useful for treatment of gonococcal infections in Kenya. Continued molecular surveillance based on larger sample size is required so as to: a) monitor the emergence and spread of ceftriaxone and azithromycin resistance since both drugs are the dual therapy currently recommended by the Kenyan National Guidelines for treatment of gonococcal infections, b) understand the effects of both efflux pumps and altered porins on antibiotic resistance in Kenyan *N. gonorrhoeae* isolates.

## Conclusion

The observed high penicillin and tetracycline resistance in the analyzed Kenyan gonococci is mainly mediated by plasmid-borne *blaTEM,* and *tetM* genes in addition to chromosomal modifications with the African type PPNG, TEM1 β-lactamase and American TetM determinants being the most prevalent. Consequently the ban on the use of these antibiotics for the treatment of gonococcal infections should continue. Asian type PPNG and Dutch TetM determinant, which are less described in the African gonococci, are present in gonococci from the studied Kenyan regions.

## Data Availability

The datasets supporting the conclusions made in this article are included within the document. The datasets are also available from the corresponding author on reasonable request. Sequence reads generated in this study are linked to NCBI BioProjects: PRJNA481622 and PRJNA590515.

## Abbreviations

β-lactamase: Beta lactamase
EUCAST: European committee on antimicrobial susceptibility testing
ESBL: Extended broad spectrum β-lactamase
GC: *Neisseria gonorrhoeae*
KEMRI: Kenya medical research institute
SERU: Scientific and ethics review unit,
WRAIR: Walter reed army institute of research
HSPB: Human subject protection board,
MIC: Minimum inhibitory concentration,
NCBI: National centre for biotechnology information
PPNG: Penicillinase Producing *Neisseria gonorrhoeae*
RAST: Rapid Annotation using subsystem technology
